# Sex and Pubertal Differences in the Type 1 Interferon Pathway Associate With Both X Chromosome Number and Serum Sex Hormone Concentration

**DOI:** 10.3389/fimmu.2018.03167

**Published:** 2019-01-15

**Authors:** Kate Webb, Hannah Peckham, Anna Radziszewska, Madhvi Menon, Paola Oliveri, Fraser Simpson, Claire T. Deakin, Sophie Lee, Coziana Ciurtin, Gary Butler, Lucy R. Wedderburn, Yiannis Ioannou

**Affiliations:** ^1^Arthritis Research UK Centre for Adolescent Rheumatology at UCL, ULCH and GOSH, London, United Kingdom; ^2^Division of Medicine, Centre for Rheumatology, UCL, London, United Kingdom; ^3^Department of Genetics, Evolution and Environment, Nanostring Facility, UCL, London, United Kingdom; ^4^NIHR Biomedical Research Centre at GOSH, London, United Kingdom; ^5^III Programme UCL GOS Institute for Child Health, London, United Kingdom; ^6^Centre for Applied Statistics Courses, Great Ormond Street Institute of Child Health, UCL, London, United Kingdom; ^7^Department of Paediatric and Adolescent Endocrinology, UCLH and Great Ormond Street Institute of Child Health, UCL, London, United Kingdom; ^8^Gender Identity Development Service (GIDS), Tavistock and Portman NHS Foundation Trust, London, United Kingdom

**Keywords:** interferon, TLR7, immunity, sex, puberty, SLE, X Chromosome, sex hormone

## Abstract

Type 1 interferons (IFN) are an antiviral cytokine family, important in juvenile onset systemic lupus erythematosus (jSLE) which is more common in females, around puberty. We report that plasmacytoid dendritic cells (pDC) from healthy females produced more type 1 IFN after toll like receptor (TLR) 7 signaling than males, even before puberty, but that puberty itself associated with increased production of type 1 IFN. A unique human model allows us to show that this was related to X chromosome number, and serum testosterone concentration, in a manner which differed depending on the number of X chromosomes present. In addition, we have showed that pDC were more activated in females overall, and immune cell *TLR7* gene expression was higher in females after puberty. Therefore, sex hormones and X chromosome number were associated individually and interactively with the type 1 IFN response, which contributes to our understanding of why females are more likely to develop an IFN mediated disease like jSLE after puberty.

## Introduction

Females have an increased immune response to viral infection and vaccination compared to males, but show an increased risk of developing certain autoimmune diseases, such as jSLE (1–4). Similar to viral infection, jSLE is distinguished by a type 1 interferon (IFN) gene signature, and is more common in females after puberty (5–7). Therefore, the effects of sex and puberty on the IFN response are important in understanding the pathogenesis of type 1 IFN-mediated diseases like jSLE. Sex and pubertal development are neglected variables in immunology research.

Within the innate immune system, plasmacytoid dendritic cells (pDC) are the chief producers of type I IFNs (8, 9) and constitutively express toll like receptors (TLR) 7 and 9 in endo-lysosomes. TLR are responsible for sensing viral and endogenous nucleic acids and trigger the release of type 1 IFNs, notably IFNα. This results in the expression of IFN stimulated genes (ISG) in immune cells leading to potent anti-viral effector functions (10). TLR7 is responsible for the sensing of single stranded RNA, whereas TLR9 senses DNA with a CpG motif (11, 12). Nucleic acid sensing, type 1 IFN production and ISG expression are a formidable and tightly regulated cascade of events.

It has been shown that TLR7-induced IFNα production is higher in females (13–16). It is unknown whether this sex difference exists in childhood, or whether it changes with puberty. It is also not known whether sex or pubertal differences exist in the percentage of pDC in peripheral blood mononuclear cells (PBMC), pDC activation, or the immune cell gene expression of *TLR7* or *TLR9*.

The X chromosome contains a large number of immune-associated genes, including *TLR7* (17–19). Studies on sex differences within the immune system have investigated sex hormone concentration or X chromosome number individually (18, 20–22). Humanized mouse models suggest that both variables may associate with type 1 IFN production (22). In humans, it is not possible to separately assess these variables, as X chromosome number and sex hormone environment are inherently linked. In this study pre- and post-pubertal young people, transgender young people on cross-sex hormone therapy and young people with Turner's syndrome (TUS) provide an experimental group, which uncouples the traditional correlation between X chromosome number and sex hormone environment. This human model reveals individual and interactive contributions of X chromosome number and serum sex hormone to the type 1 IFN response. These novel insights contribute to our understanding of the sex differences in the innate immune response, and help us understand some of the sex and pubertal bias in an IFN mediated disease like jSLE.

## Methods

### Patients and Controls

Young volunteers were recruited in a cross sectional manner, and informed consent was sought with Research Ethics Committee approval REC11/0101. Healthy participants older than 12 years were recruited from the local community at science outreach events. Healthy participants less than 12 years old were only recruited if blood was being taken for an unrelated clinical indication (e.g., surgery for routine, non-inflammatory procedures). One young healthy female (13 years old) was excluded with an oestradiol concentration of 2106 pmol/L which was beyond the range of healthy non-pregnant adult females. Transgender young people on cross-sex hormone therapy (i.e., testosterone in those born phenotypically female and oestradiol in those born phenotypically male) and young women with TUS were recruited from endocrinology clinics. Three transgender males (birth females) were excluded as they had testosterone concentrations of greater than 60 nmol/L which is beyond the normal range of a healthy age matched, cis-gender birth males. This could be due to the injection of testosterone on the day of sampling, or the use of a higher dose than recommended. Young women with TUS all displayed the characteristic phenotype associated with a single X chromosome, although they had varying genotypes (Supplementary Table 1). Young people with low disease activity jSLE, (SLE disease activity index -SLEDAI <5) and a steroid dose <15 mg/day (median dose 3 mg/day) were recruited from rheumatology clinics (Supplementary Table 2). Exclusion criteria included acute or chronic illness and concurrent medication (including oral contraceptive). Upon consent, demographic data, clinical data, and peripheral blood was collected. In addition, participants completed a pubertal self-assessment questionnaire, based upon established pubertal developmental phases (23, 24). These phases are adapted from the Tanner staging system(25), but offer only 3 categories (Pre-Puberty, In-Puberty and Completed-Puberty) and are simpler and less invasive than the Tanner system. They have been adopted for standard pubertal screening in the United Kingdom (26). They are summarized in Supplementary Tables 3, 4. Ages of participants are summarized in Supplementary Table 5.

### Human Cell Isolation

Serum and PBMC were separated from heparinized blood, by Ficoll gradient separation and immediately cryopreserved.

### Cell Culture

PBMC were cultured in RPMI 1640 containing L-glutamine and NaHCO_3_ (Sigma-Aldrich) supplemented with 10% FCS (Biosera) and 100 IU/μg/ml penicillin/streptomycin (Sigma-Aldrich) in 96-well U-bottom plates. One million cells were each stimulated with TLR7 agonist, R848 (Invivogen-tlrl-r848) at 1ug/ml or TLR9 agonist, CpGODN2216 (CpG) (Invivogen tlrl-2216) at 1μM or left unstimulated (in RPMI) for 20 h. Brefeldin A (Sigma B7651) was added to the unstimulated and R848-stimulated cells at 0 h, and added to the CpG-stimulated cells at 16 h. To test for variation in experiments, a single healthy volunteer was used in each experiment. In addition, where experiments were performed on different days, experiment batch was used as a control variable in each regression model in sensitivity analysis, to account for variation, and found not to be influential.

### Flow Cytometry

Upon thawing, one million cells were immediately stained with a panel of antibodies to assess *ex vivo* phenotype (RRID given when available): Tetherin-APC (BioLegend Cat# 348410, RRID:AB_2067121); CD86-PeCy7 (BioLegend Cat# 305422, RRID:AB_2074981); CD4-BV711(BioLegend Cat# 317439, RRID:AB_11219404); CD303-PE (BioLegend Cat# 354203, RRID:AB_11125176; CD56-PerCP Cy5.5 (BioLegend Cat# 318322, RRID:AB_893389); CD11c-BV421 (BioLegend Cat# 301627,RRID:AB_10898313); CD8 V500 (BD Cat# 561618); CD14 BUV 737 (BD. Cat# 564444); CD19-BUV 395 (BD Cat# 563549); CD3-BUV 805 (BD Cat #565515). Gates were set using isotype control samples (Gating strategy: Supplementary Figure 1). After stimulation, cells were washed, and stained with surface antibodies: Lineage -CD3, CD14, CD19, CD20, CD56- FITC (BioLegend Cat# 348701, RRID:AB_10644012); BDCA2 PE (BioLegend Cat# 354203, RRID:AB_11125176); CD123 PECy7 (Biolegend Cat# 306009). Following incubation, cells were washed, fixed, permeabilized (Ebioscience IC fix 88-8824-00) and stained for detection of intracellular IFNα (IFNα APC, Miltenyi Biotec, Cat#130-092-602). Samples were run on an LSRII flow cytometer (BD) and the data were analyzed using Flowjo v. 10. To test for variation in experiments, a single healthy volunteer was used in each experiment. In addition, where experiments were performed on different days, experiment batch was used as a control variable in each regression model in sensitivity analysis, to account for variation, and found not to be influential.

### Cell Culture Supernatant Assay

One million PBMC were separately stimulated with R848, CpG or left unstimulated in medium without the addition of Brefeldin A. After 20 h, supernatant was removed and cryopreserved. Supernatants were analyzed by bead-based multiplex assay (Luminex-Thermofisher) at the UMC Utrecht facility for the concentrations of IFNα, IFNβ, and TNFα. These data were not normally distributed and the natural log of IFNα, IFNβ, and TNFα concentration was used when these were analyzed by linear regression.

### Nanostring Plexset Technology

Separately, depending on availability of PBMC, RNA was extracted from cells using Picopure RNA isolation (Thermofisher, KIT0214) immediately upon thawing, or after 20 h stimulation with IFNα2b (Sigma, SRP4595) at 1000 IU/ml. RNA gene expression was quantified using Nanostring Plexset technology, whereby digital barcodes hybridize to oligonucleotide probes to assess for expression of pre-chosen genes, after correction to house-keeping genes (*POL2RA, G6PD, SDHA*) and inbuilt positive and negative controls (27). A panel of genes was chosen (Supplementary Table 6) and analyzed by linear regression, applying the Benjamini-Hochberg method of correction for multiple testing for the significance value of each coefficient.

### Liquid Chromatography/Mass Spectrometry

In addition, serum was analyzed using liquid chromatography/mass spectrometry (LCMS) for the serum concentrations of testosterone and oestradiol.

### Statistical Analysis

Data were analyzed using linear regression, *t*-tests or Mann Whitney U tests as statistically appropriate. Where necessary, the Benjamini Hochberg or Bonferroni methods of correction for multiple hypothesis testing were applied. SPSS software was used for statistical analysis (v.24). Graphs were created with SPSS or Biovinci graph software (v.1.3). The numbers of samples in each subgroup with available data are summarized in Supplementary Table 7. If outliers were present, sensitivity analysis was performed, and if the outliers were not influential, they were included in the analysis.

## Results

### pDC in Healthy Females Expressed More Surface CD86 and Tetherin Than Males, Regardless of Puberty

It was first investigated whether the pDC percentage, activation or surface expression of the anti-viral protein, tetherin differed with sex or pubertal phase in healthy volunteers. All full regression models are summarized in Supplementary Table 8.

**Figure 1:**
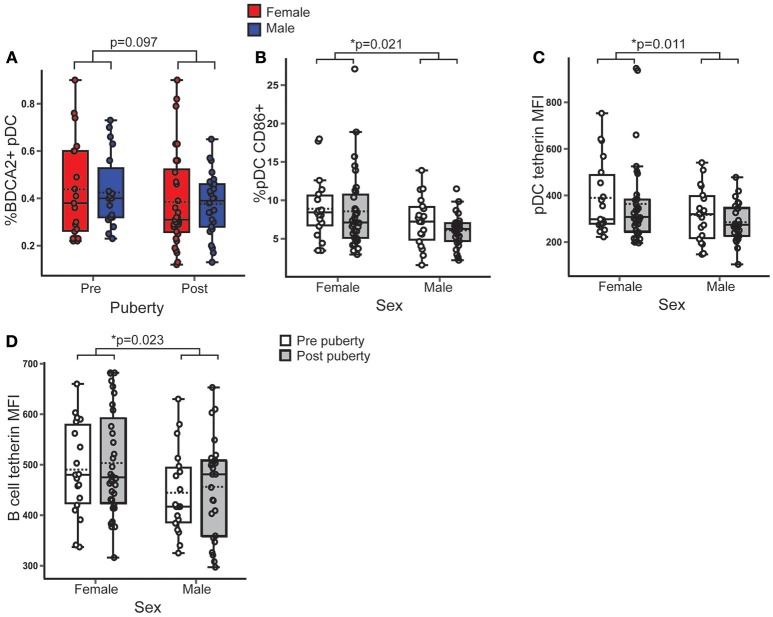
Female pDC expressed more surface CD86 and tetherin than males, regardless of puberty. Healthy volunteer PBMC were assessed by flow cytometry (*n* = 106). *p*-values represent the significance of the coefficient the variable shown as estimated by linear regression after correcting for the other variable in the model (sex or puberty). **(A)** %BDCA2+ pDC did not differ with pubertal phase (*p* = 0.097). **(B)** In females, a higher % pDC expressed CD86 than in males (*p* = 0.021). **(C,D)** Females had a higher tetherin expression in pDC (*p* = 0.011) and B cells (*p* = 0.023) than males. *p < 0.05.

There was no difference in the percentage of BDCA2^+^ pDC in PBMC between sexes (*B* = 0.005; *p* = 0.889; 95% CI = −0.064, 0.073) or pubertal phases (*B* = −0.059, *p* = 0.097; 95% CI = −0.129, 0.011) (Figure 1A).

CD86 is a T cell costimulatory molecule and marker of activation on pDC, that is upregulated by TLR associated signaling (28). Females, on average, had 1.9% more pDC expressing CD86 than males (*B* = 1.88; *p* = 0.021; 95% CI = 0.294, 3.465), with no significant difference between pubertal phases (Figure 1B) indicating that female pDC were more activated than males, regardless of puberty.

Tetherin is a type 1 IFN inducible antiviral membrane protein constitutively expressed by pDC and B cells (29, 30). *Ex vivo* surface expression of tetherin on pDC (*B* = 75.767; *p* = 0.011; 95% CI = 17.568, 133.965) (Figure 1C) and B cells (*B* = 54.156; *p* = 0.023; 95% CI = 6.229, 84.082) (Figure 1D) was higher in females than males, with no association with pubertal phase.

Therefore, although there was no difference in the percentage of pDC, healthy female pDC were more activated and expressed more of an anti-viral surface marker than males, regardless of pubertal development, implying an inherently increased anti-viral pDC phenotype in females.

### CD86 Expression by pDC Was Higher When Two X Chromosomes Were Present, Regardless of Serum Sex Hormone Concentration

**Figure 2:**
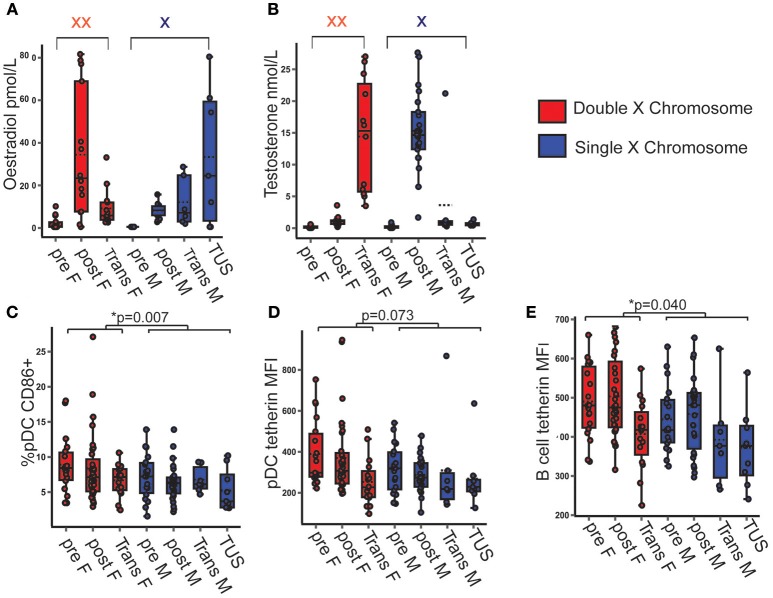
pDC CD86 and tetherin expression were higher when two X chromosomes were present, regardless of serum testosterone and oestradiol concentration. **(A,B)** Serum hormone levels in pre- and post-pubertal healthy, transgender and TUS volunteers allowed for a full spectrum of hormone concentration upon the background of one or two X chromosomes (*n* = 135). *p*-values represent the significance of the coefficient the variable shown as estimated by linear regression after correcting for the other variables in the model (X chromosome number, serum testosterone, and oestradiol). **(C)** If two X chromosomes were present, there was a higher %pDC expressing CD86 (*p* = 0.007). **(D)** The presence of two X chromosomes showed a non-significant trend toward a higher pDC tetherin expression (*p* = 0.073). **(E)** B cell tetherin expression was higher in the presence of two X chromosomes (*p* = 0.040). pre F, Pre- pubertal female; post F, Post-pubertal female; Trans F, Transgender birth female; pre M, Pre-pubertal male; post M, Post-pubertal male; Trans M, transgender birth male; TUS, turners syndrome female. *p < 0.05.

After observing that female pDC were more activated and expressed more tetherin than those of males, it was next investigated whether the number of X chromosomes present or the differences in serum sex hormone concentrations accounted for these differences. Transgender young people on cross-sex hormone therapy, and young people with TUS were included to build a model with a full spectrum of serum concentrations of each sex hormone on a background of a single or double X chromosome (Figures 2A,B). All full regression models analyzing the associations between each dependent variable and X chromosome number and serum sex hormone are summarized in Supplementary Table 9.

The presence of two X chromosomes was associated with 2.4% more pDC expressing surface CD86 on average (*B* = 2.406; *p* = 0.007; 95% CI = 0.660, 4.152) after controlling for serum sex hormone concentrations (Figure 2C).

Although not significant, volunteers with two X chromosomes trended toward a higher pDC surface tetherin expression (*B* = 68.082; *p* = 0.073; 95% CI = −6.618, 142.783) and a significantly higher B cell tetherin expression (*B* = 45.874; *p* = 0.040; 95% CI = 2.249, 89.498) controlling for serum sex hormone concentration (Figures 2D,E).

Therefore, this model allows us to show that the increased pDC activation and expression of the anti-viral protein tetherin seen in females associated with the number of X chromosomes present, and not with variations of serum sex hormone *in vivo*.

### TLR7 Induced pDC IFNα Production was Higher in Females, Regardless of Puberty, and Higher After Puberty, Regardless of Sex

After demonstrating that pDC in females, showed a more anti-viral phenotype, regardless of puberty, it was investigated whether pDC IFNα production differed with sex or pubertal phase in healthy volunteers. Full models are given in Supplementary Table 8.

**Figure 3:**
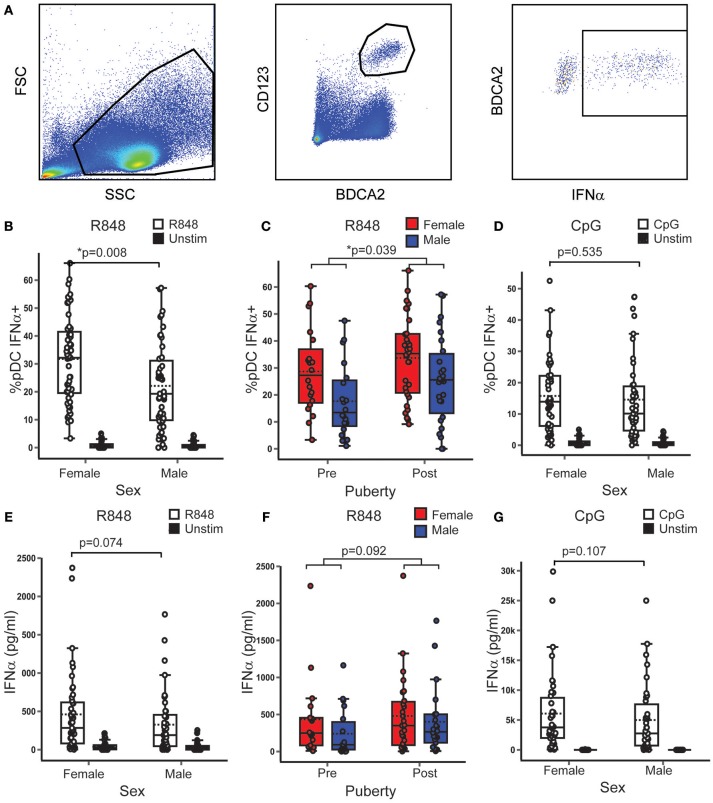
In healthy volunteers, TLR7, and not TLR9 induced pDC IFNα production was higher in females and after puberty. **(A)** After stimulation with R848 or CpG, PBMC from healthy volunteers were assessed by flow cytometry as shown. *p*-values represent the significance of the coefficient the variable shown as estimated by linear regression after correcting for the other variable in the model (sex or puberty). **(B,C)** Upon R848 stimulation, females had a higher % pDC IFNα+ (*p* = 0.008) than males, and post-pubertal volunteers had a higher % pDC IFNα+ (*p* = 0.039), than pre-pubertal volunteers (*n* = 109). **(D)** After CpG stimulation, there was no sex difference in the % pDC IFNα+ (*p* = 0.535, *n* = 90). **(E,F)** After R848 stimulation, the same trend toward higher PBMC production of IFNα in females (*p* = 0.074) and post-pubertal volunteers (*p* = 0.092) was seen as in the pDC specific experiment, although this did not reach significance (*n* = 86). **(G)** No sex differences were seen in PBMC IFNα production after CpG stimulation (*p* = 0.107, *n* = 74). *p < 0.05.

Controlling for pubertal phase, females had on average 8.33% more pDC producing IFNα after R848 (TLR7 ligand) stimulation than males (*B* = 8.334; *p* = 0.008; 95% CI = 2.214, 14.454) (Figures 3A,B). In addition, the model showed that, when controlling for sex, post-pubertal volunteers had on average 6.5% more pDC producing IFNα after R848 stimulation than the pre-pubertal group (*B* = 6.569; *p* = 0.039; 95% CI = 0.336, 12.802) (Figure 3C). After CpG (TLR9 ligand) stimulation, there were no significant associations between the percentage of pDC producing IFNα and either sex (Figure 3D) or pubertal phase.

It was confirmed that the majority of cells which produced IFNα when PBMC were stimulated with R848 were pDC (Supplementary Figure 2). To confirm the pDC specific flow cytometry findings, PBMC production of IFNα, IFNβ, and TNFα into culture supernatants was measured after stimulation with the same agonists. These variables were transformed using the natural log function to account for the non-linear relationship between the outcome and predictor variables. These showed a similar trend to the pDC specific experiments, but did not reach the same statistical significance. In this sample, after R848 stimulation, males produced 51% less IFNα than females (ExpB = 0.49; *p* = 0.074; 95% CI = 0.22, 2.01) (Figure 3E) and post-pubertal volunteers produced almost double the amount of IFNα than the pre-pubertal group (ExpB = 1.94; *p* = 0.092; 95% CI = 0.89, 4.25) (Figure 3F). There was no association with sex or pubertal phase and the total PBMC production of IFNβ or TNFα after stimulation with R848.

When PBMC were stimulated with CpG, there were no associations between supernatant PBMC production of IFNα (Figure 3G), IFNβ or TNFα with sex or pubertal phase.

Therefore it was observed that in healthy young people, regardless of pubertal development, females had a higher percentage of pDC producing IFNα than males after TLR7 stimulation. In addition to this, regardless of sex, post-pubertal volunteers had a higher percentage of pDC producing IFNα after TLR7 stimulation than pre-pubertal volunteers. This effect was specific to TLR7 and IFNα. Therefore, female sex and puberty independently associated with the TLR7 induced production of IFNα.

### X Chromosome Number and Serum Testosterone Concentration Were Associated With TLR7-Induced IFNα Production

After demonstrating a higher pDC TLR7 associated IFNα production in females and post-pubertal volunteers, it was investigated whether the number of X chromosomes or differences in sex hormone concentrations accounted for these differences. Transgender volunteers and volunteers with TUS were included to investigate the associations between pDC-derived IFNα production, X chromosome number and serum sex hormone.

**Figure 4:**
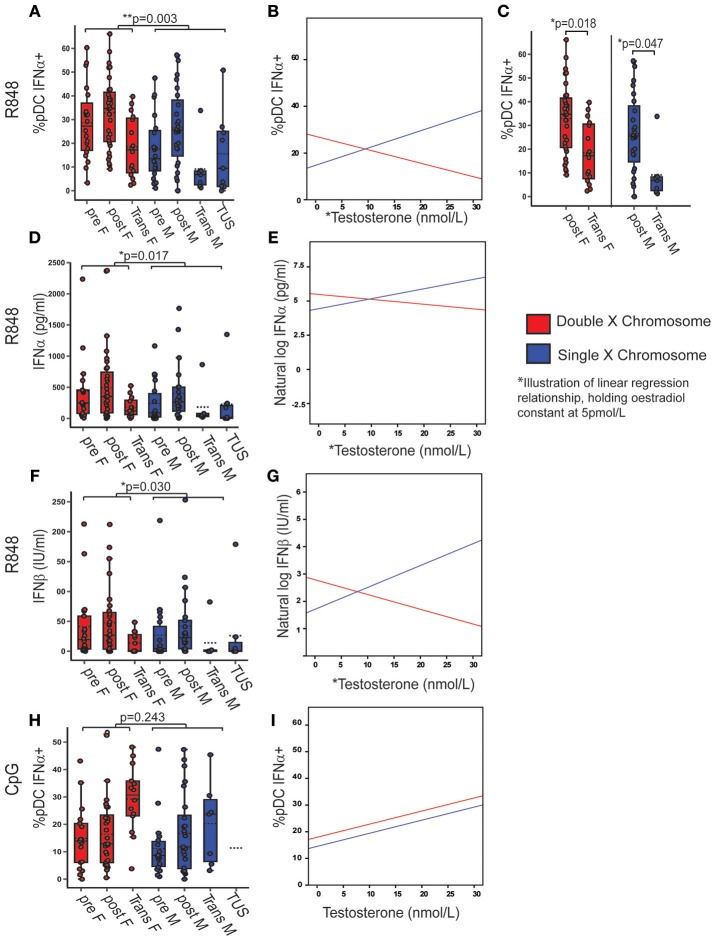
X chromosome number and serum testosterone concentration was associated with TLR7 induced IFNα production. Pre- and post-pubertal healthy, transgender, and TUS volunteers were analyzed (*n* = 128), *p*-values represent the significance of the coefficient the variable shown as estimated by linear regression after correcting for the other variables in the model (X chromosome number, serum testosterone, and oestradiol). **(A)** If two X chromosomes were present, a higher % pDC produced IFNα after R848 stimulation (*p* = 0.003). **(B)** % pDC IFNα+ after R848 stimulation associated with serum testosterone (*p* = 0.008) with a significant interaction term between X chromosome number and serum testosterone (*p* = 0.002) as illustrated, holding oestradiol constant at 5pmol/L. **(C)** Transgender birth females (*p* = 0.018) and males (*p* = 0.047) both had a lower %pDC IFNα+ after R848 stimulation than their chromosomal counterparts when analyzed by ANOVA with *post hoc* analysis. **(D–G)** After R848 stimulation, if two X chromosomes were present, PBMC produced more IFNα (*p* = 0.017) and IFNβ (*p* = 0.03) with a significant interaction between testosterone and X chromosome number (*p* = 0.028; *p* = 0.019 respectively). **(H,I)** After CpG stimulation, % pDC IFNα+ did not associate with X chromosome number (*p* = 0.243) but did associate with serum testosterone concentration (*p* = 0.007). pre F, Pre-pubertal female; post F, Post- pubertal female; Trans F, Transgender birth female; pre M, Pre-pubertal male; post M, Post-pubertal male; Trans M, transgender birth male; TUS, turners syndrome female. *p < 0.05; **p < 0.005.

If two X chromosomes were present, regardless of sex hormone concentrations, there were on average 12.41% more pDC producing IFNα after R848 stimulation (*B* = 12.407; *p* = 0.003; 95% CI = 4.434, 20.380) compared to if one X was present (Figure 4A) (Full model Supplementary Table 10). The best fit regression model included an association with serum testosterone (*B* = 0.740; *p* = 0.008; 95% CI = 0.197, 1.284) and a significant interaction term between X chromosome number and serum testosterone (*B* = 1.315; *p* = 0.002; 95% CI = −2.144, −0.486). This implies that there is a positive association with the percentage of pDC producing IFNα after R848 stimulation and testosterone in the presence of one X chromosome, and a negative association in the presence of two X chromosomes, after controlling for oestradiol (an illustration of this regression model, when oestradiol is held constant at 5 pmol/L, is given in Figure 4B). By this model, it would be expected that females (double X) with more testosterone and males (single X) with less testosterone would both have fewer pDC producing IFNα after TLR7 stimulation. Indeed when this was investigated by ANOVA with a Bonferroni adjusted *post-hoc* test, it was confirmed that there was a lower percentage of IFNα-producing pDC in transgender males (birth female-two X chromosomes; high testosterone) compared to healthy females (mean difference = 15.23; *p* = 0.018; 95% CI = 1.75–28.72) and transgender females (birth males-one X chromosome, low testosterone) when compared to healthy males (mean difference = 17.41; *p* = 0.047; 95% CI = 0.16–34.66) (Figure 4C). Overall, the model accounted for 10% of the variability in the percentage of pDC capable of producing IFNα after R848 stimulation (adjusted *r*^2^ = 0.101, *p* = 0.012).

The same model was fitted to the PBMC production of IFNα or IFNβ in supernatant, after R848 stimulation, in order to test the pDC-specific findings above. These outcomes were transformed using a natural log transformation to account for their non-linear relationship with the predictors. After R848 stimulation, if two X chromosomes were present, there was on average 2.8 times more IFNα (ExpB = 2.84; *p* = 0.017; 95% CI = 1.21–6.67) (Figure 4D) and 2.9 times more IFNβ (ExpB = 2.94; *p* = 0.030; 95% CI = 1.115–7.791) (Figure 4F) produced than if one X chromosome was present. Serum testosterone was significantly associated with increased PBMC IFNα (ExpB= 1.08; *p*= 0.032; 95% CI= 1.01–1.14) and IFNβ production (ExpB = 1.08; *p* = 0.030; 95% CI = 1.01–1.16). There were significant interaction terms between serum testosterone and X chromosome number with IFNα (ExpB = 0.90; *p* = 0.027; 95% CI = 0.80–0.98) (Figure 4E) and IFNβ (ExpB = 0.875; *p* = 0.019; 95% CI = 0.78, 0.98) (Figure 4G) production, as there had been in the pDC specific model, again implying a positive association with testosterone in the presence of one X chromosome and a negative association in the presence of two. The supernatant production of TNFα after R848 stimulation did not associate with serum sex hormone concentration or X chromosome number

Upon CpG stimulation, the percentage of pDC producing IFNα did not significantly differ with X chromosome number (*p* = 0.243) (Figure 4H, Supplementary Table 9) but was associated with testosterone (*B* = 0.49; *p* = 0.007; 95% CI = 0.14, 0.85) (Figure 4I). There were no associations between the total PBMC production of IFNα, IFNβ, or TNFα and sex or pubertal phase after CpG stimulation.

Therefore if two X chromosomes were present, regardless of serum sex hormone, pDC produced more type 1 IFN after TLR7 stimulation specifically. In addition, serum testosterone concentration associated with the production of type 1 IFN after TLR7 stimulation differently, depending on the number of X chromosomes present.

### PBMC TLR7 Gene Expression Was Significantly Higher in Post-Pubertal Females

After demonstrating that female pDC were more activated, expressed more tetherin and produced more IFNα after TLR7 signaling, it was next investigated, whether the PBMC gene expression of *TLR7, TLR9*, and ISG differed with sex or pubertal phase in healthy volunteers. Full models are given in Supplementary Table 8.

**Figure 5:**
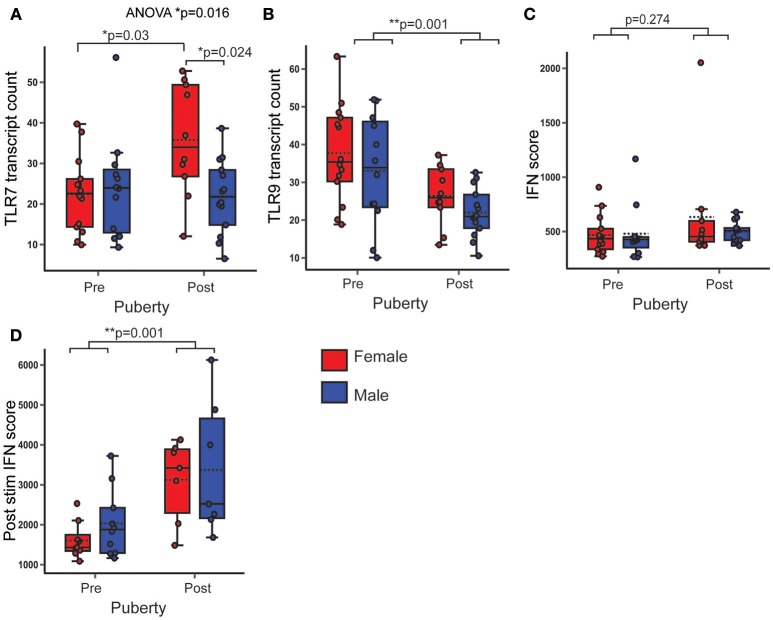
PBMC gene expression of TLR7 was higher in post-pubertal females. In healthy volunteers, PBMC gene expression was assessed by Nanostring and normalized gene transcript counts are shown (*n* = 50). *p*-values represent significance of the coefficient for the variables shown after correcting for the other variable (sex or puberty). **(A)** There was an overall difference in *TLR7* expression (*p* = 0.016). After *post hoc* testing with Bonferroni correction, post-pubertal females had a higher *TLR7* expression than post-pubertal males (*p* = 0.024) and pre-pubertal females (*p* = 0.03) **(B)** PBMC *TLR9* gene expression was lower in post-pubertal volunteers. **(C)** There were no pubertal (*p* = 0.274) differences in the PBMC IFN score. **(D)** After stimulation with IFNα, the IFN score (post stim IFN score) was significantly higher in post pubertal volunteers (*p* = 0.001, *n* = 33). *p < 0.05; **p < 0.005.

PBMC *TLR7* gene expression was not significantly associated with pubertal phase, but was non-significantly higher in females (*B* = 5.749; *p* = 0.091; 95% CI = −0.952, 12.45). An ANOVA with *post hoc* Bonferroni correction revealed that *TLR7* gene expression was significantly higher in post-pubertal females, when compared to all other groups (*p* = 0.016) (Figure 5A). PBMC *TLR9* gene expression was significantly lower in post-pubertal volunteers (*B* = −11.156; *p* = 0.001; 95% CI = −17.398, −4.913), with no significant sex difference (Figure 5B).

There were no significant sex or pubertal differences in the *ex vivo* IFN score-calculated from the expression of five ISG (*MX1*+*MCP1*+*ISG15*+*IFTIT1*+*BST2/5* = IFN score, Figure 5C). After pre-stimulation with IFNα however, there was a higher IFN score, on average, in the post pubertal volunteers (*B* = 1428.677; *p* = 0.001; 95% CI = 699.565, 2157.789), with no significant sex difference (Figure 5D).

Therefore post pubertal females had an increased gene expression of *TLR7* after puberty, whereas *TLR9* expression was decreased in the post-pubertal volunteers with no sex difference. There was a markedly increased ability of PBMC to upregulate ISG in response to IFNα stimulation after puberty.

Of note, one 16 year old healthy female had a markedly higher IFN score. Clinical data were reviewed which indicated no evidence to suggest a concurrent viral infection. Sensitivity analysis was performed and as this outlier was not influential, it was elected to include the data in the analysis.

### PBMC TLR7 Gene Expression Was Not Associated With X Chromosome Number or Serum Sex Hormone

In order to assess whether X chromosome number or serum sex hormone concentration accounted for the increase in *TLR7* expression in post-pubertal females, the model was again extended to include transgender volunteers and those with Turner's syndrome (Supplementary Table 9).

**Figure 6:**
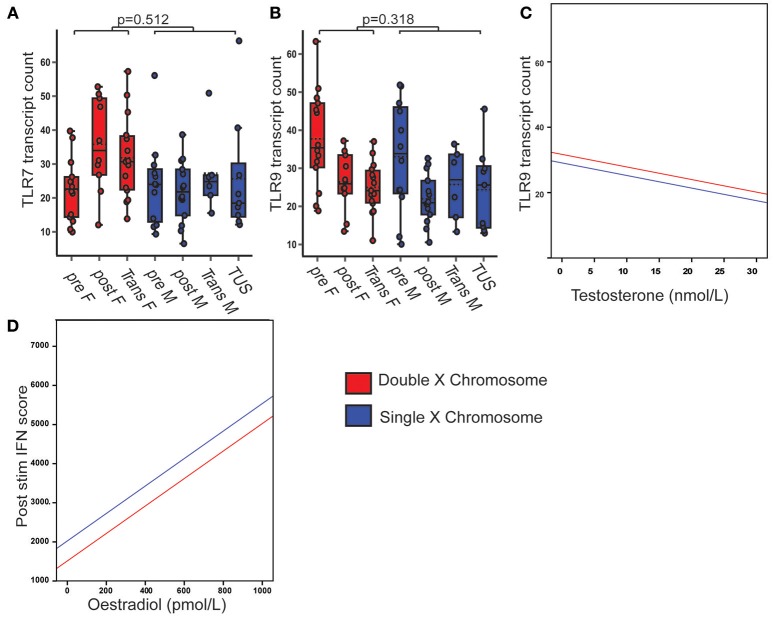
PBMC TLR7 gene expression did not associate with X chromosome number or serum sex hormone. Pre- and post-pubertal healthy, transgender and TUS volunteers were included (*n* = 77), *p*-values represent the significance of the coefficient the variable shown as estimated by linear regression after correcting for the other variables in the model (X chromosome number, serum testosterone, and oestradiol). **(A)** PBMC TLR7 gene expression did not differ with X chromosome number (*p* = 0.512). **(B,C)** PBMC TLR 9 gene expression did not differ with X chromosome number (*p* = 0.318), but was negatively associated with serum testosterone concentration (*p* = 0.014). **(D)** After cells were pre-stimulated with IFNα, IFN score was significantly associated with serum oestradiol (*p* = 0.009, *n* = 58).

PBMC *TLR7* gene expression was not significantly associated with X chromosome number (Figure 6A), serum testosterone or oestradiol concentrations. PBMC *TLR9* gene expression was not significantly associated with X chromosome number, but was negatively associated with serum testosterone concentration (*B* = −0.385; *p* = 0.014; CI = −0.691, −0.079) (Figures 6B,C).

The IFN score in IFNα pre-stimulated cells was associated with serum oestradiol concentration, regardless of testosterone or X chromosome number (*B* = 3.511; *p* = 0.009; 95% CI = 0.972, 6.049) (Figure 6D).

### PBMC TLR7 Expression Was Higher in Males With jSLE

jSLE has a type 1 IFN signature, a strong female predominance, and manifests more commonly at puberty. Samples from healthy, post-pubertal young people were compared with those from age matched post- pubertal young people with jSLE (by Mann-Whitney (MW) U or *t*-test, depending on the distribution of the outcome, and corrected for multiple testing with Benjamini Hochberg method). Patients with jSLE and low disease activity were specifically chosen as disease activity is known to correlate with ISG expression and may act as a confounder (Supplementary Table 2) (5).

**Figure 7:**
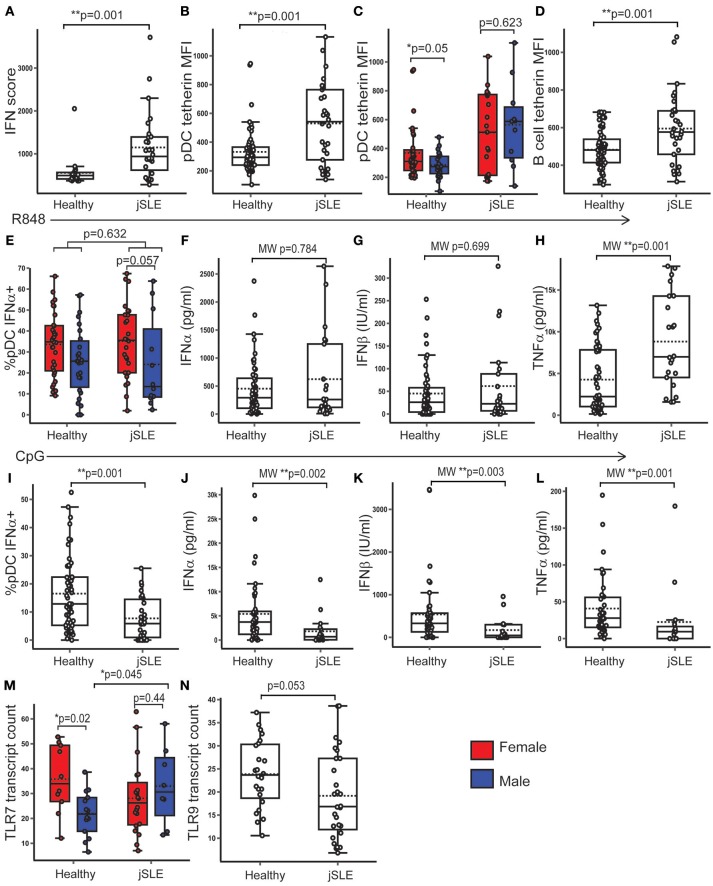
PBMC TLR7 gene expression was higher in males with jSLE. Healthy volunteers and young people with jSLE were included. *p*-values represent *t*-tests unless data was not normally distributed, when a Mann Whitney U test was done as indicated by “MW.” **(A)** PBMC IFN score was higher in jSLE (*p* = 0.001, *n* = 79). **(B)** pDC tetherin expression was higher in jSLE (*p* = 0.001, *n* = 136). **(C)** The sex difference in pDC tetherin expression seen in healthy volunteers (*p* = 0.05) was lost in those with jSLE (*p* = 0.623). **(D)** B cell tetherin expression was higher in jSLE (*p* = 0.004, *n* = 136). **(E)** After R848 stimulation, there was no significant difference in the %pDC producing IFNα in jSLE (*p* = 0.632, *n* = 138). Females with jSLE had a trend toward a higher % pDC producing IFNα than males with jSLE (*p* = 0.057). **(F–H)** There were no differences in jSLE in the amount of IFNα (*p* = 0.784) or IFNβ (*p* = 0.699) produced by PBMC in supernatant after R848 stimulation, but there was more TNFα produced in jSLE (*p* = 0.001, *n* = 108). **(I–L)** After stimulation with CpG, there was a reduction in the production of all cytokines in patients with jSLE (*n* = 92). **(M)** PBMC TLR7 gene expression was not different overall in jSLE (*p* = 0.636, *n* = 79) although the sex difference was lost in jSLE (*p* = 0.440). Males with jSLE had a higher PBMC TLR7 gene expression (*p* = 0.045) than healthy males. **(N)** There was a trend toward decreased PBMC gene expression of TLR9 in jSLE (*p* = 0.053, *n* = 43). *p < 0.05; **p < 0.005.

There were no significant differences in serum testosterone or oestradiol concentration between healthy young people and those with jSLE. As expected, young people with jSLE had a higher PBMC IFN score than healthy young people (*t*-test: *p* = 0.001; 95% CI = 251.27, 1066.80) (Figure 7A). Both pDC (*t*-test: *p* = 0.001; 95% CI = 111.18, 292.39) and B cells (*t*-test: *p* = 0.001; 95% CI = 50.64, 174.92) from patients with jSLE displayed an increased expression of surface tetherin compared to healthy young people (Figures 7B,D). In healthy volunteers, pDC from females tended to express more tetherin (*t*-test: *p* = 0.05; 95% CI = −0.25, 155.35), whereas this sex difference was lost in young people with jSLE (*t*-test: *p* = 0.623; 95% CI = −270.84, 164.97) (Figure 7C). There was no significant difference in CD86 expression by pDC (*t*-test: *p* = 0.10; 95% CI = −0.33, 3.71) or B cells (*t*-test: *p* = 0.231; 95% CI = −0.62, 2.51) in jSLE.

After R848 stimulation, the percentage of IFNα-producing pDC (*t*-test; *p* = 0.632; 95% CI = −8.94, 5.46) and the amount of IFNα (MW: p = 0.784) or IFNβ (MW: p = 0.699) produced by PBMC was not significantly different in jSLE compared to healthy volunteers (Figures 7E–G). Females with jSLE still had a higher percentage of pDC producing IFNα after R848 stimulation than males although this did not reach statistical significance (*t*-test: *p* = 0.057; 95% CI = −0.39, 25.5) (Figure 7E). The amount of TNFα produced by PBMC upon R848 stimulation was higher in jSLE than in healthy volunteers (MW; *p* = 0.001), with no sex differences (Figure 7H). Upon CpG stimulation, production of all measured cytokines was decreased in patients with jSLE. There were fewer pDC producing IFNα (*t*-test: *p* = 0.001; 95% CI = −14.00, −4.03) and less IFNα (MW: *p* = 0.002), IFNβ (MW: *p* = 0.003) and TNFα (MW: *p* = 0.001) produced by PBMC than in healthy controls after CpG stimulation (Figures 7I–L).

As reported above, in healthy, post-pubertal young people, PBMC gene expression of *TLR7* was significantly higher in post-pubertal females compared to males. Although there was no overall difference in *TLR7* expression between healthy volunteers and those with jSLE (*t*-test: *p* = 0.646; 95% CI = −9.625, 6.02), this sex difference was not present in volunteers with jSLE (*t*-test: *p* = 0.440; 95% CI = −17.69, 7.92). On further analysis, males with jSLE had a higher gene expression of *TLR7* in PBMC compared to healthy males (*t*-test: *p* = 0.045; 95% CI = −21.91, −0.27) (Figure 7M). As an increase in ISG expression is expected in jSLE, PBMC from healthy controls were stimulated with IFNα and gene expression measured which confirmed that *TLR7* was not IFN inducible in healthy volunteers (Supplementary Figure 3). There was trend toward a decreased PBMC *TLR9* gene expression in volunteers with jSLE (*t*-test: *p* = 0.053; 95% CI = −0.058, 9.43), despite *TLR9* behaving as an IFN inducible gene in healthy volunteers (Figure 7N).

Therefore, it is noted that TLR9 induced IFNα production and gene expression was decreased in young people with low disease activity jSLE as compared to healthy volunteers. In contrast, TLR7 induced IFNα production or gene expression was not changed in low disease activity jSLE.

## Discussion

In these data, female sex, pubertal development and X chromosome number were associated with pDC activation, TLR7-mediated IFN production and IFN inducible protein and gene expression. The unique young volunteers in this study have allowed for new insights into how X chromosome number and serum sex hormone contribute individually and interactively to sex and pubertal differences in the type 1 IFN mediated innate immune system which may explain some of the female predominance seen in a type 1 IFN mediated immune disease like jSLE.

These data are the first to observe that in the resting state, pDC are more activated and have a more “anti-viral” or IFN-driven phenotype in females, regardless of puberty. In the model that included transgender young people and young females with TUS, it was demonstrated that this related to the presence of two X chromosomes, regardless of sex hormone concentration. This implies that the presence of two X chromosomes associates inherently with pDC that are more activated and display a more anti-viral phenotype.

It has been previously shown that a higher percentage of pDC in adult and neonatal females produced IFNα after TLR7 stimulation (13–15, 31). It has not been clear whether this is due to differences in sex hormone concentration or X chromosome number in humans. Adults and neonates [due to the mini-puberty of infancy (32)] have relatively high concentration of both sex hormones, and it was not previously known whether this difference was present in childhood, where sex hormone concentration is low in both sexes. We show for the first time that females (including children) had a significantly and substantially higher percentage of IFNα-producing pDC than males upon TLR7 stimulation, regardless of pubertal phase. Puberty however, did individually associate with a higher percentage of IFNα-producing pDC after TLR7 stimulation, regardless of sex. Remarkably, in healthy volunteers, these essentially “demographic” variables accounted for 10% of the variability seen in the TLR7 induced production of IFNα, a potent and strictly regulated cytokine.

Furthermore, these unique, young, human volunteers allowed us to show that the percentage of pDC producing IFNα after TLR7 stimulation was significantly and substantially higher if two X chromosomes were present, when controlling for the effect of sex hormone. Interestingly, serum testosterone, and not oestradiol, associated with TLR7-induced pDC IFNα and the direction of this association differed depending on the number of X chromosomes present. This is the first time that this bi-directional association has been observed and is largely due to the inclusion of transgender volunteers in the study who demonstrated this effect. These data suggest that in females specifically, testosterone may be a candidate for further investigation to potentially modulate the TLR7 mediated pDC IFN response.

pDC only represent 0.1–0.9% of total PBMC, although they have been shown to produce approximately 98% of the IFNα produced by PBMC (6). Due to small blood volume and cell numbers obtainable in children and adolescents, it was not possible to separate sufficient pDC to assess for the pDC-specific total amount of IFNα produced in supernatant. Therefore, the amount of IFNα produced in supernatant by total PBMC after stimulation with R848 was measured as confirmation of the pDC-specific findings. This confirmed the same trend toward higher production in females and post-pubertal groups, and showed the same significant association with X chromosome number, and the bi-directional relationship with testosterone.

Although it has not been possible to investigate for the individual effects of serum sex hormone and X chromosome number in humans before, mouse models have been designed to unlink these variables. These demonstrated that pDC from human progenitor cells transplanted into humanized mice produced more IFNα upon TLR7 activation, if they originated from a female human, regardless of the sex of the recipient mouse, suggesting an inherent role of X chromosome number in TLR7 mediated IFNα production (22). The current data provide a human model which confirms this observation.

R848 is an agonist of both TLR7 and TLR8 (33). Although the effects of TLR8 stimulation cannot be definitively excluded from these data, pDC are known to constitutively express TLR7 and not TLR8, and therefore the results most likely represent TLR7 stimulation (34, 35). The indirect effect of TLR8 on other cell types, however, cannot be excluded.

TLR7 related production of IFNα or IFNβ was not significantly different in jSLE. TLR7-induced IFNα production has been reported to be the same or increased in SLE, and has been associated with disease activity (6, 36, 37) which is perhaps why a difference was not seen in these patients with low disease activity. Interestingly, after TLR7 stimulation, there was a significantly higher production of TNFα in jSLE. This is in contrast to TLR9 stimulation, where the production of all cytokines was significantly lower in jSLE patients, as has been reported before (38, 39). All of the patients with jSLE were treated with hydroxychloroquine, which has the potential to interfere with TLR9 and TLR7 induced cytokine production in a manner that is not fully understood *in vivo* (40, 41). Hydroxychloroquine is almost universally used in jSLE and often started at the first diagnosis, therefore we are unlikely to be able to perform these experiments in its absence.

Although PBMC gene expression of *TLR7* and *TLR9* did not directly correlate with the functional pDC findings, there were some interesting observations. *TLR7* gene expression was shown to be significantly higher after puberty in females, which did not relate to either sex hormone concentration or X chromosome number. We cannot exclude whether other pubertal hormones, such as luteinizing hormone or follicular stimulating hormone, or other non-hormonal parameters may be associated with the increase in *TLR7* expression after puberty in females. Recent data in adults has shown that *TLR7*, which is encoded on the X chromosome, may escape X inactivation in a significant portion of B cells, monocytes and pDC (21). Future work is needed to assess whether puberty in females is associated with increased bi-allelic expression of *TLR7*. Conversely, PBMC *TLR9* gene expression was lower in the post-pubertal group and associated with serum testosterone concentration. Although no sex or pubertal differences were seen in the baseline expression of ISG in PBMC, there was a markedly enhanced upregulation of ISGs in response to IFNα stimulation in post pubertal volunteers, which was associated with serum oestradiol concentration.

In the BXSB male mouse model, with a predisposition to SLE, the presence of a translocated *TLR7* allele (y linked autoimmune accelerator -*yaa*), and an over-expression of *TLR7*, potently associates with disease development (42, 43). We report that males with jSLE displayed a significantly higher PBMC gene expression of *TLR7* than healthy male volunteers, suggesting that *TLR7* over-expression may be a contributing factor for jSLE development in human males.

PBMC *TLR9* gene expression was lower in the post-pubertal group and associated with serum testosterone concentration. There was a trend toward a lower expression of *TLR9* in volunteers with jSLE. Emerging data suggests that that *TLR9* gene expression may be protective against the development of SLE, whereas *TLR7* expression mediates inflammation, IFNα production and disease progression (42–46). The combination of a decrease in *TLR9* expression after puberty, associated with testosterone, along with an increase in *TLR7* expression in post-pubertal females, may contribute to the described increased susceptibility among females to develop jSLE upon sexual maturity (7).

There is emerging evidence that an increased type 1 IFN response may underpin the development of SLE in susceptible individuals (47). Although no sex or pubertal differences were seen in the baseline expression of ISG in PBMC, enhanced upregulation of ISGs in response to IFNα stimulation was observed in samples taken from subjects after puberty, which was associated with serum oestradiol. This, along with the observation that female sex and pubertal phase associate with the increased production of type 1 IFN, contributes to our understanding of why females are more prone to develop jSLE around puberty.

A limitation of the present study is the cross-sectional design and lack of longitudinal data across both puberty and gender transition. Although patients with jSLE specifically had a low disease activity and minimal steroid dosage, none were truly treatment naïve. The nature of this large cohort of unique young people is that the data are associative in nature, and further functional work is needed to investigate the direct mechanisms underlying the novel associations reported here.

In conclusion, these unique human data demonstrate complex interplays between oestradiol, testosterone and X chromosome number. In summary they show that female sex and pubertal development associate with an increased production of and response to type 1 IFN in a manner that may underpin the increased prevalence of jSLE in females after puberty.

## Data Availability Statement

Datasets are available on request: The raw data supporting the conclusions of this manuscript will be made available by the authors, without undue reservation, to any qualified researcher.

## Ethics Statement

This study was carried out in accordance with the recommendations of North Harrow Research Ethics Committee REC11/0101, with written informed consent from all subjects. All subjects gave written informed consent in accordance with the Declaration of Helsinki. The protocol was approved by the North Harrow Research Ethics Committee.

## Author Contributions

KW, LW, YI, MM, AR, and GB contributed conception and design of the study. KW, AR, and HP recruited patients and collected specimens. KW organized the database. KW and AR performed the experiments. FS and PO performed the Nanostring experiment. KW and SL performed the statistical analysis. CD and SL checked the statistical analysis. KW wrote the first draft of the manuscript. AR wrote a section of the manuscript. All authors contributed to manuscript revision, read and approved the submitted version.

### Conflict of Interest Statement

The authors declare that the research was conducted in the absence of any commercial or financial relationships that could be construed as a potential conflict of interest.
