# Supplementary material for: Sex and Pubertal Differences in the Type 1 Interferon Pathway Associate With Both X Chromosome Number and Serum Sex Hormone Concentration
Source: Front Immunol. 2019 Jan 15;9:3167. doi: 10.3389/fimmu.2018.03167 (PMC6345344; doi:10.3389/fimmu.2018.03167)
Supplement: Supplementary file 1 [file Table_1.DOCX]

Supplementary Material

**Sex and pubertal differences in the type 1 interferon pathway associate with both X chromosome number and serum sex hormone concentration.**

**Kate Webb*, Hannah Peckham, Anna Radziszewska, Madhvi Menon, Paola Oliveri, Fraser Simpson, Claire T. Deakin , Sophie Lee , Coziana Ciurtin, Gary Butler , Lucy R. Wedderburn, Yiannis Ioannou.**

*** Correspondence:** Kate Webb, kate.webb@ucl.ac.uk

**Supplementary Data**

Supplementary material

Supplementary table 1. Turners syndrome patients characteristics.

| Age | Therapy | Genetic diagnosis |
| --- | --- | --- |
| 15.3 | Oestradiol | Turner variant, deletion of chromosome Xq21.3 |
| 19.6 | Oestradiol and growth hormone | Turner mosaicism (45,X/46,X,idic(X)(p11.2) |
| 11.4 | Growth hormone | Turner syndrome 45,X |
| 15.2 | Oestradiol and growth hormone | Turner syndrome 45,X |
| 18.2 | Thyroxine and oestradiol | Turner 45,X |
| 15.9 | Growth hormone | Turner mosaic (45,X/46,XX) |
| 15.8 | Oestradiol and growth hormone | 45,X Turner |
| 16.7 | Vitamin D | Turner mosaic 45,X/47,XXX |
| 13.8 | Growth hormone and vitamin D | Turner mosaic 47,XXX/45,X |

|  | Female (n=26) | | Male (n=13) | |
| --- | --- | --- | --- | --- |
|  | Median | Range | Median | Range |
| Age | 16.67 | 14.7-17.7 | 18.2 | 16.4-20.7 |
| Average SLEDAI | 2 | 0-12 | 2 | 0-24 |
| CRP (mg/L) | .60 | 0.06-10.7 | 1.00 | 0.6-18 |
| WCC (x10^9^/L) | 4.97 | 1.0-10.0 | 4.97 | 2.75-7.00 |
| C3 (g/L) | 1.07 | 0.61-1.77 | .93 | 0.33-1.30 |
| C4 (g/L) | .19 | 0.06-0.43 | .14 | 0-0.27 |
| ESR (mm/hr.) | 11 | 2.0-29.0 | 10 | 0-47 |
| dsDNA (IU/ml) | 26 | 0-687 | 62 | 0-2827 |
| Median daily dose | 3 | 0-15 | 0 | 0-15 |

Supplementary Table 2. Characteristics jSLE patients

**SLEDAI=Systemic lupus erythematosus disease activity index; CRP=C reactive protein; WCC-=White cell count; C3/4=complement ¾; ESR=Erythrocyte sedimentation rate; dSDNA =antidouble stranded DNA antibody titre*GE=gene expression; TUS =Turner syndrome*

Supplementary Table 3. Male pubertal phases (RCPCH Growth Chart(1))

| Pre-puberty(Tanner stage 1) | In puberty (Tanner stage 2-3) | Completed puberty (Tanner stages 4-5) |
| --- | --- | --- |
| If **both** of the following: | If **any** of the following: | If **any** of the following |
| No signs of pubertal development  High voice | Early pubic or armpit hair growth  Enlargement of testes or penis | Voice fully broken  Moustache and early facial hair growth  Adult size of penis and axillary hair |

Supplementary Table 4. Female pubertal phases (RCPCH Growth Chart(1)).

| Pre-puberty(Tanner stage 1) | In puberty (Tanner stage 2-3) | Completed puberty (Tanner stages 4-5) |
| --- | --- | --- |
| If: | If **any** of the following: | If **all** of the following |
| No signs of pubertal development | Any breast enlargement so long as nipples enlarged  Any pubic or axillary hair | Starting periods (menarche) with breast, pubic and axillary hair development. |

Supplementary Table 5. Volunteers by age, sex and pubertal phase.

| Volunteers | Pubertal phase | n | Age | | |
| --- | --- | --- | --- | --- | --- |
|  |  |  | Median | Min | Max |
| Healthy female | Pre pubertal | 20 | 8.7 | 6.3 | 12.01 |
|  | Post pubertal | 31 | 17.04 | 12.34 | 18.32 |
| Healthy male | Pre pubertal | 20 | 8.68 | 6.46 | 12.43 |
|  | Post pubertal | 28 | 17.08 | 13.45 | 18.8 |
| Trans male (Birth female) | Post pubertal | 13 | 18.83 | 17.63 | 19.46 |
| Trans female (Birth male) | Post pubertal | 7 | 18.61 | 17.3 | 19.53 |
| jSLE female | Post pubertal | 26 | 16.67 | 14.7 | 17.7 |
| jSLE male | Post Pubertal | 13 | 18.2 | 16.4 | 20.7 |

Supplementary Table 6. Nanostring Plexset pre-designed gene panel

| ***Gene name*** | | ***Function*** |
| --- | --- | --- |
| *MX1* | *Interferon induced binding protein 1* | *Interferon inducible* |
| *BST2* | *Tetherin* | *Interferon inducible* |
| *IFIT1* | *Interferon induced protein with tetra repeats 1* | *Interferon inducible* |
| *ISG15* | *interferon stimulated gene 15* | *Interferon inducible* |
| *MCP1* | *monocyte chemotactic protein 1* | *Interferon inducible* |
| *IRF5* | *Interferon regulatory factor 5* | *IRF* |
| *IRF7* | *Interferon regulatory factor 7* | *IRF* |
| *TLR7* | *Toll like receptor 7* | *TLR* |
| *TLR9* | *Toll like receptor 9* | *TLR* |
| *TNFR6* | *Fas* | *cell death* |
| *TRAIL* | *TNF related apoptosis inducing ligand* | *cell death* |
| *L1* | *LINE-1* | *Endogenous*  *retroelement* |
| *DDX41* | *Dead box helicase 41* | *cytoplasmic viral*  *sensing* |
| *MDA5* | *Melanoma differentiated protein 5* | *cytoplasmic viral*  *sensing* |
| *RIG1* | *Retinoid inducible gene 1* | *cytoplasmic viral*  *sensing* |
| *MAVS* | *Mitochondrial antiviral signaling protein* | *cytoplasmic viral*  *sensing* |
| *TROVE2* | *Ro60* | *Ro 60* |
| *ESR1* | *estrogen receptor alpha* | *hormone receptor* |
| *ESR2* | *estrogen receptor beta* | *hormone receptor* |
| *AR* | *androgen receptor* | *hormone receptor* |
| *PGR* | *progesterone receptor* | *hormone receptor* |
| *G6PD* | *housekeeper* |  |
| *POL2RA* | *housekeeper* |  |
| *SDHA* | *housekeeper* |  |

Supplementary Table 7: Numbers of samples with data for each measure

|  | Sex | Puberty | Ex vivo  flow | R848  %pDC  IFNα+ | CPG  %pDC  IFNα+ | R848 IFNα  (pg/ml) | CPG IFNα  (pg/ml) | Ex vivo  GE | Post stim  GE | Oestradiol | Testosterone |
| --- | --- | --- | --- | --- | --- | --- | --- | --- | --- | --- | --- |
| Healthy | F | Pre | 19 | 20 | 18 | 18 | 15 | 14 | 9 | 16 | 19 |
|  |  | Post | 37 | 31 | 28 | 27 | 24 | 10 | 7 | 14 | 20 |
|  | M | Pre | 19 | 20 | 19 | 19 | 17 | 12 | 10 | 13 | 18 |
|  |  | Post | 31 | 28 | 25 | 22 | 18 | 14 | 7 | 17 | 22 |
| TUS | F | Post | 9 | 9 | 9 | 8 | 6 | 9 | 6 | 7 | 7 |
| Trans (Birth  sex) | F | Post | 13 | 13 | 11 | 11 | 6 | 12 | 7 | 11 | 12 |
|  | M | Post | 7 | 7 | 7 | 6 | 4 | 6 | 5 | 6 | 7 |
| jSLE | F | Post | 19 | 26 | 22 | 16 | 13 | 20 | 9 | 18 | 19 |
|  | M | Post | 11 | 13 | 9 | 6 | 5 | 9 | 1 | 8 | 8 |

|  | **Model: y=intercept+B_1_*Sex+B_2_*Puberty** | | | | | | | | | | |
| --- | --- | --- | --- | --- | --- | --- | --- | --- | --- | --- | --- |
| Investigation | Dependant variable | Model predictive value | Model significance | Independent Variables | | | | | | | |
|  |  |  |  | Sex (F=1; M=0) | | | | Puberty(Pre=0; Post=1) | | | |
|  |  | Adjusted r2 | p | B_1_ | p | 95% CI | | B_2_ | p | 95% CI | |
| % pDC in PBMC | %pDC | 0.009 | 0.249 | 0.005 | 0.889 | -0.064 | 0.073 | -0.059 | 0.097 | -0.129 | 0.011 |
| pDC CD86 expression | %pDC CD86 | 0.041 | 0.052 | 1.88 | 0.021 | 0.294 | 3.465 | -0.671 | 0.413 | -2.291 | 0.949 |
| Tetherin expression | pDC tetherin | 0.053 | 0.029 | 75.767 | 0.011 | 17.568 | 133.965 | -27.304 | 0.364 | -86.757 | 32.149 |
|  | B cell tetherin | 0.039 | 0.059 | 54.156 | 0.023 | 6.229 | 84.082 | 13.238 | 0.51 | -26.527 | 53.003 |
| TLR7 stim | %pDC IFNα+ | 0.092 | 0.004 | 8.334 | 0.008 | 2.214 | 14.454 | 6.569 | 0.039 | 0.336 | 12.802 |
|  | ln(IFNα) (pg/ml) | 0.074 | 0.042 | 0.*495a* | 0.074 | *0.22a* | *2.01a* | 1.*948a* | 0.092 | 0.*894a* | 4.*250a* |
|  | ln(IFNβ)(IU/ml) | 0.039 | 0.192 | *-*0.*635a* | 0.216 | *-1.309a* | *-0.308a* | 1.*589a* | 0.21 | 0.*766a* | 3.*299a* |
|  | ln(TNFα)(pg/ml) | -0.019 | 0.804 | *-*1.*013a* | 0.668 | *-1.075a* | *-0.955a* | 1.*016a* | 0.599 | 0.*957a* | 1.*079a* |
| TLR9 Stim | %pDC IFNα+ | 0.005 | 0.299 | 1.601 | .535 | -3.509 | 6.711 | 3.681 | .162 | -1.510 | 8.872 |
|  | ln(IFNα) (pg/ml) | 0.04 | 0.231 | *-*0.*512a* | 0.107 | *-1.16a* | *-0.225a* | 0.*736a* | 0.462 | 0.*322a* | 1.*681a* |
|  | ln(IFNβ) (IU/ml) | 0.003 | 0.332 | *-*0.*482a* | 0.145 | *-1.294a* | *-0.18a* | 1.*059a* | 0.909 | 0.*392a* | 2.*863a* |
|  | ln(TNFα)(pg/ml) | -0.017 | 0.688 | *-*0.*82a* | 0.39 | *-1.296a* | *-0.518a* | 0.*991a* | 0.39 | 0.*624a* | 1.*573a* |
| Gene expression | *TLR7* | 0.055 | 0.099 | 5.749 | 0.091 | -0.956 | 12.450 | 5.246 | 0.122 | -1.455 | 11.947 |
|  | *TLR9* | 0.23 | 0.001 | 4.566 | 0.148 | -1.676 | 10.809 | -11.156 | 0.001 | -17.398 | -4.913 |
|  | IFN score | -0.008 | 0.455 | 58.776 | 0.461 | -100.47 | 218.023 | 87.641 | 0.274 | -71.606 | 246.888 |
|  | Post stim IFN Score | 0.316 | 0.001 | -351.992 | 0.327 | -1073.01 | 369.033 | 1428.677 | 0.001 | 699.565 | 2157.789 |

Supplementary Table 8: Summary of all regression model to assess for associations between variables in type 1 IFN pathway, with sex and puberty. Benjamini Hochberg method of multiple correction was applied within each investigation type. Healthy pre and post pubertal volunteers only were included. arepresents expB.

Supplementary Table 9: Summary of all regression models to assess for associations between variables in type 1 IFN pathway with X

chromosome number, serum testosterone concentration and oestradiol concentration. Healthy pre- and post-pubertal, transgender

volunteers and volunteers with Turners syndrome were included. (*The most parsimonious model for TLR9 gene expression did not include

serum oestradiol concentration)

|  | **Model: y=intercept+B_1_*X Chromosome number+B_2_*Testosterone+B_3_*Oestradiol** | | | | | | | | | | | | | | |
| --- | --- | --- | --- | --- | --- | --- | --- | --- | --- | --- | --- | --- | --- | --- | --- |
|  |  | Model predictive value | Model significance | Independent Variables | | | | | | | | | | | |
|  | Dependant variable | Adjusted R^2^ | p |  |  |  |  |  |  |  |  |  |  |  |  |
|  |  |  |  | X chromosome number (1=0;2=1) | | | | Testosterone | | | | Oestradiol | | | |
|  |  |  |  | B_1_ | p | 95% CI | | B_2_ | p | 95% CI | | B_3_ | p | 95% CI | |
| pDC CD86  expression | %pDC CD86+ | 0.083 | 0.017 | 2.406 | 0.007 | 0.66 | 4.152 | -0.59 | 0.05 | -0.17 | 0.05 | 0.01 | 0.664 | -0.01 | 0.01 |
| Tetherin expression | pDC tetherin | 0.044 | 0.082 | 68.082 | 0.073 | -6.62 | 142.783 | -3.54 | 0.13 | -8.11 | 1.04 | -0.08 | 0.465 | -0.30 | 0.14 |
|  | B cell tetherin | 0.031 | 0.132 | 45.874 | 0.04 | 2.25 | 89.498 | -1.09 | 0.42 | -3.76 | 1.58 | -0.22 | 0.731 | -0.15 | 0.11 |
| TLR9 Stim | %pDC IFNα+ | 0.069 | 0.04 | 3.423 | 0.243 | -2.38 | 9.22 | 0.49 | 0.007 | 0.14 | 0.85 | 0.01 | 0.666 | -0.01 | 0.02 |
| Gene expression | *TLR7* | -0.052 | 0.929 | 2.497 | 0.512 | -5.091 | 10.084 | 0.021 | 0.920 | -.398 | .441 | 0.001 | 0.926 | -0.020 | 0.022 |
|  | *TLR9* | 0.078 | 0.024 | 2.693 | 0.318 | -2.654 | 8.039 | -0.385 | 0.014 | -0.691 | -0.079 | * |  |  |  |
|  | Stim IFN Score | 0.247 | 0.015 | -522.391 | 0.189 | -1318.9 | 274.12 | 29.62 | 0.25 | -22.42 | 81.65 | 3.511 | 0.009 | 0.972 | 6.05 |

Supplementary Table 10: Full linear regression model assessing for associations between X chromosome number; sex hormone and IFNα

production after R848 stimulation in healthy (pre- and post-puberty), Turner and transgender volunteers

| **Model: y=Intercept+B_1_*X chromosome number+B_2_*Testosterone+B_3_*Testosterone*X chromosome number +B_4_*Oestradiol** | | | | | | | | | | | | |
| --- | --- | --- | --- | --- | --- | --- | --- | --- | --- | --- | --- | --- |
| **Independent Variables** | Dependent variables | | | | | | | | | | | |
|  |  | | | | | | | | | | | |
|  | **% pDC IFNα+**  **Adjusted r^2^=0.101; p=0.012** | | | | **ln IFNα (pg/ml) Adjusted r2=0.101;**  **p=0.024** | | | | **ln IFNβ (pg/ml) Adjusted r2=0.049;**  **p=0.115** | | | |
|  | B | p | 95% CI | | ExpB | p | 95% CI | | ExpB | p | 95% CI | |
| X chromosome number (1=0; 2=1) | 12.41 | 0.003** | 4.43 | 20.38 | 2.84 | 0.017* | 1.21 | 6.67 | 2.95 | 0.03* | 1.12 | 7.79 |
| Testosterone nmol/L | 0.74 | 0.008* | 0.20 | 1.28 | 1.08 | 0.032* | 1.01 | 1.14 | 1.08 | 0.03* | 1.01 | 1.16 |
| Test/X interaction | -1.32 | 0.002** | -2.14 | -0.49 | 0.90 | 0.027* | 0.81 | 0.99 | 0.87 | 0.019* | 0.78 | 0.98 |
| Oestradiol pmol/L | 0.01 | 0.57 | -0.01 | 0.03 | 0.99 | 0.05 | 1.00 | 1.00 | 1.00 | 0.63 | 1 | 1 |

Supplementary Figure 1. Ex-vivo PBMC gating strategy.

a.Ex vivo cells were gated as PBMC by size and granularity after excluding live/dead and doublet cells (not shown). PBMC were then further gated as pDC (BDCA2+, CD123+), monocytes (CD14+ vs CD11c+); lymphocytes(CD3+ T cells : CD4+ vs CD8+; CD19+ B cells).b.CD86 expression was measured on ex-vivo antigen presenting cells by flow cytometry and gates set as demonstrated using isotype controls.


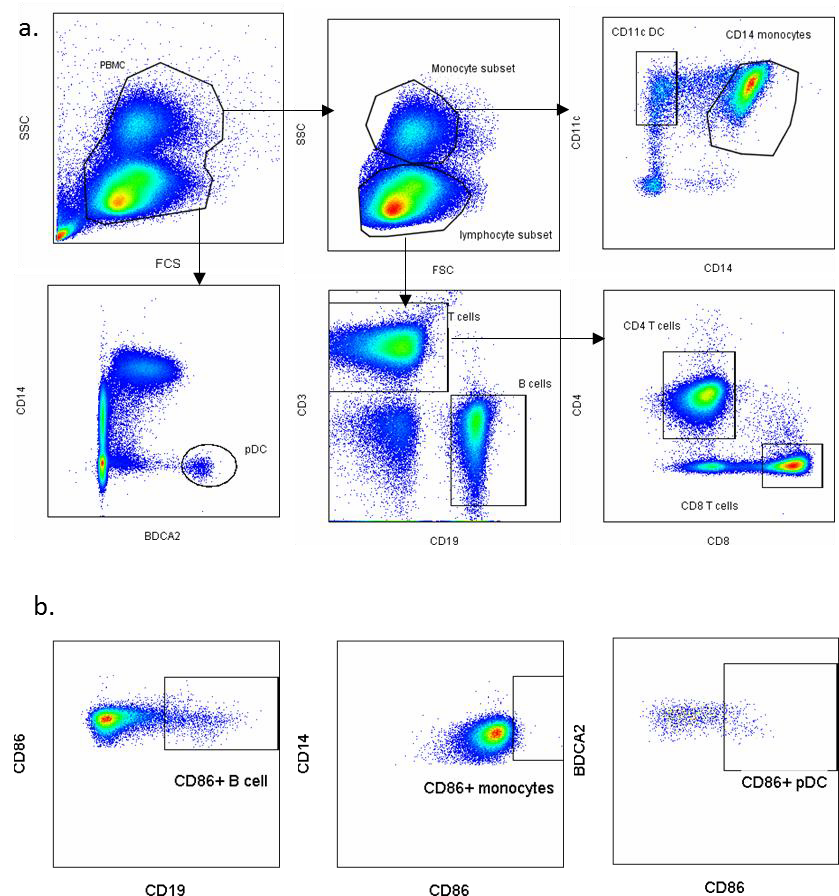


Supplementary Figure 2. pDC were the main producers of IFNα

a. PBMC were stimulated with R848 and the production of IFNα was assessed in CD3+ T cells, CD14+ monocytes, CD19+ B cells and BDCA2+ pDC. The majority of IFNα+ cells were confirmed to be pDC.


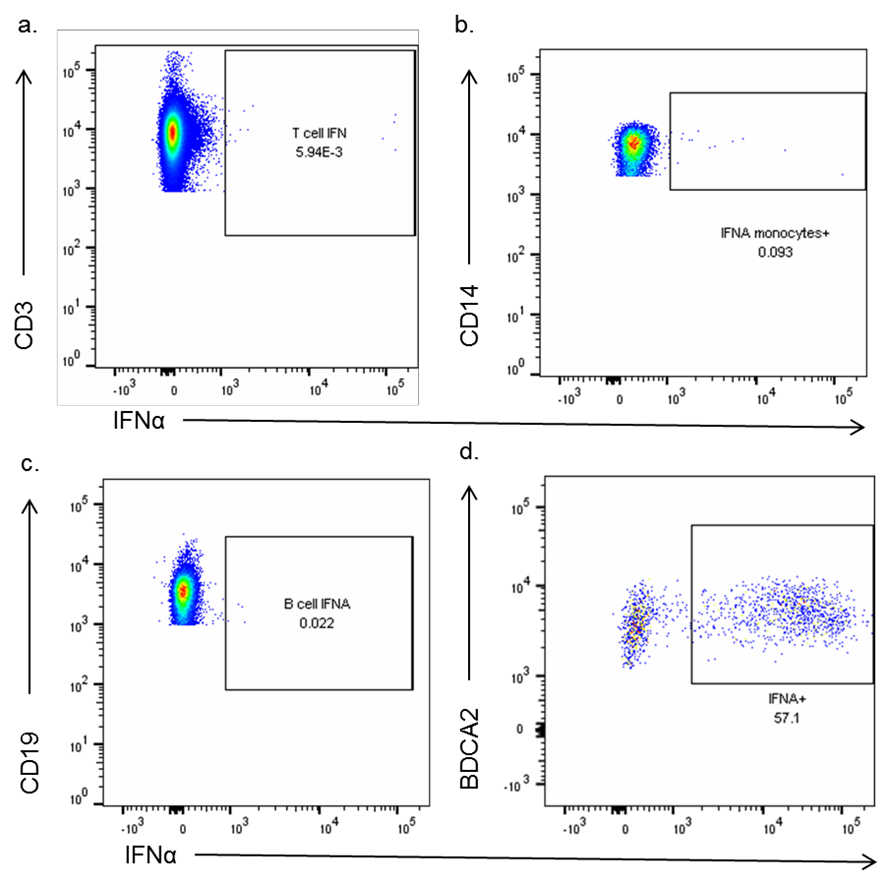


Supplementary Figure 3. Interferon inducible genes

In order to confirm which genes were IFN inducible, PBMC RNA gene expression was analysed ex vivo or after stimulation with IFNα for 20 hours by Nanostring and compared. Significance values were corrected for multiple testing using Bonferroni method. *p-values<0.003 indicates genes which are IFN inducible.

| ***IRF7** | **0.000000** |
| --- | --- |
| ***MDA5** | **0.000000** |
| ***MX1** | **0.000000** |
| ***RIG-1** | **0.000000** |
| ***MAVS** | **0.000000** |
| ***MCP-1** | **0.000000** |
| ***ISG15** | **0.000000** |
| ***BST2** | **0.000000** |
| ***IFIT-1** | **0.000001** |
| ***L1** | **0.000003** |
| ***TNFR6** | **0.000024** |
| ***TLR9** | **0.000058** |
| ***TROVE2** | **0.001632** |
| **DDX41** | **0.003687** |
| **TLR7** | **0.018325** |
| **TRAIL** | **0.066654** |
| **IRF5** | **0.067258** |

1. Growth charts | RCPCH 2018 [Available from: <https://www.rcpch.ac.uk/resources/growth-charts>.
